# Social and clinical determinants of preferences and their achievement at the end of life: prospective cohort study of older adults receiving palliative care in three countries

**DOI:** 10.1186/s12877-017-0648-4

**Published:** 2017-11-23

**Authors:** Irene J. Higginson, Barbara A. Daveson, R. Sean Morrison, Deokhee Yi, Diane Meier, Melinda Smith, Karen Ryan, Regina McQuillan, Bridget M. Johnston, Charles Normand, on behalf of BuildCARE, Emma Bennett, Francesca Cooper, Barbara Daveson, Susanne de Wolf-Linder, Mendwas Dzingina, Clare Ellis-Smith, Catherine Evans, Taja Ferguson, Lesley Henson, Irene J. Higginson, Bridget Johnston, Paramjote Kaler, Pauline Kane, Lara Klass, Peter Lawlor, Paul McCrone, Regina McQuillan, Diane Meier, Susan Molony, Sean Morrison, Fliss Murtagh, Charles Normand, Caty Pannell, Steve Pantilat, Anastasia Reison, Karen Ryan, Lucy Selman, Melinda Smith, Katy Tobin, Rowena Vohora, Gao Wei, Deokhee Yi

**Affiliations:** 1 Cicely Saunders Institute Of Palliative Care, Policy & Rehabilitation, King’s College London, and King’s College Hospital, Bessemer Road, London, SE5 9PJ UK; 2 Department of Geriatrics and Palliative Medicine, Mount Sinai School of Medicine, Icahn School of Medicine at Mount Sinai, One Gustave L. Levy Place, New York, NY 10029-6574 USA; 3 Mater Misericordiae Hospital, Eccles Street, Dublin 7, Ireland; 4 Beaumont Hospital, Beaumont Road, Dublin 9, Ireland; 5 The Centre of Health Policy and Management, Trinity College Dublin, Room 0.21, 3-4 Foster Place, College Green, Dublin 2, Ireland

**Keywords:** Palliative care, End-of-life care, Preferences, Place of death, Home, Hospice, Hospital, Ageing, Elderly

## Abstract

**Background:**

Achieving choice is proposed as a quality marker. But little is known about what influences preferences especially among older adults. We aimed to determine and compare, across three countries, factors associated with preferences for place of death and treatment, and actual site of death.

**Methods:**

We recruited adults aged ≥65-years from hospital-based multiprofessional palliative care services in London, Dublin, New York, and followed them for >17 months. All services offered consultation on hospital wards, support for existing clinical teams, outpatient services and received funding from their National Health Service and/or relevant Insurance reimbursements. The New York service additionally had 10 inpatient beds. All worked with and referred patients to local hospices. Face-to-face interviews recorded most and least preferred place of death, treatment goal priorities, demographic and clinical information using validated questionnaires. Multivariable and multilevel analyses assessed associated factors.

**Results:**

One hundred and thirty eight older adults (64 London, 59 Dublin, 15 New York) were recruited, 110 died during follow-up. Home was the most preferred place of death (77/138, 56%) followed by inpatient palliative care/hospice units (22%). Hospital was least preferred (35/138, 25%), followed by nursing home (20%) and home (16%); hospice/palliative care unit was rarely least preferred (4%). Most respondents prioritised improving quality of life, either alone (54%), or equal with life extension (39%); few (3%) chose only life extension. There were no significant differences between countries. Main associates with home preference were: cancer diagnosis (OR 3.72, 95% CI 1.40–9.90) and living with someone (OR 2.19, 1.33–3.62). Adults with non-cancer diagnoses were more likely to prefer palliative care units (OR 2.39, 1.14–5.03). Conversely, functional independence (OR 1.05, 1.04–1.06) and valuing quality of life (OR 3.11, 2.89–3.36) were associated with dying at home. There was a mismatch between preferences and achievements – of 85 people who preferred home or a palliative care unit, 19 (25%) achieved their first preference.

**Conclusion:**

Although home is the most common first preference, it is polarising and for 16% it is the least preferred. Inpatient palliative care unit emerges as the second most preferred place, is rarely least preferred, and yet was often not achieved for those who wanted to die there. Factors affecting stated preferences and met preferences differ. Available services, notably community support and palliative care units, require expansion. Contrasting actual place of death with capacity for meeting patient and family needs may be a better quality indicator than simply ‘achieved preferences’.

**Electronic supplementary material:**

The online version of this article (10.1186/s12877-017-0648-4) contains supplementary material, which is available to authorized users.

## Background

Good health care should respond to individual views and preferences; the unique things about a person. Recent policies in many countries centre on meeting choice and preferences, such as the recent UK Choice Review [1] and the US House Bill 5555, “Personalize Your Care Act of 2016” [2]. One important component of choice in end-of-life care is place of care and death [1–3]. This is also essential for service planning – to ensure that the right services are developed to provide care, and to understand the large variations between and within countries [4–6]. Surveys and prioritisation research show that most people with advanced illness want to die at home, although an important minority prefers other locations [3, 7, 8]. However, the probability of home death diminishes with age [6]. This has important implications as, across the globe, populations are ageing rapidly, with more deaths at older ages [9]. Future projections indicate that home deaths may reduce, rather than increase [10]. Some evidence suggests home death is not preferred among older people, [11] other studies contradict this and research is scant, [3, 7] despite the fact that most people who die are aged over 65 years. As a result, several bodies have recommended a move away from measuring home death rates as a quality indicator, proposing ‘achieving a preference’ as a better quality indicator, while acknowledging that it is difficult to measure [12, 13].

A relatively large body of research has estimated factors associated with actual place of death [14, 15]. Diversely little is known about factors associated with preferences for place of death. Research in different countries suggests that preferences are constant for 70–90% [8, 16–18]. There are anecdotal reports of adults who do change their preferences and qualitative studies have found that positive and negative experiences of care can alter preference for place of death [19]. Without a better understanding of factors influencing preferences for place of death, ‘achieving a preference’ risks being a flawed and misleading quality indicator. Preferences are more nuanced than a simple ‘first choice’; understanding what is least preferred is fundamental when developing appropriate quality indicators; ‘least preferred’ may not simply be the reverse of ‘most preferred’ [20].

This study aimed to determine the associates with the most and least preferred place of death, treatment priorities and whether these are similar to or different from the associates with actual place of death across three countries with developed specialist palliative care. We focused on people aged 65 years and older because they are at highest risk of dying, rising rapidly in population, and the least studied. Secondary aims were to: describe and explore the similarities and differences between individuals and their preferences and priorities receiving palliative care in the three countries. Given the differences in culture, health care funding systems and eligibility for palliative care and hospice services we hypothesised that this might lead to differences in preferences, for example favouring treatment to increase quantity rather than quality of life, or preference for palliative or hospice care.

## Methods

### Study design and approvals

This prospective cohort study was part of the BuildCARE programme to investigate access, care experience, outcomes and their determinants for older people with advanced diseases. We recruited older adults receiving inpatient and outpatient multidisciplinary palliative care. We enrolled consecutive consenting adults aged 65 years and older from inpatient and outpatient palliative care programs and subsequently recorded information about where they died. The study received ethical approval from relevant bodies (see declarations for details). Findings are reported following STROBE and MORECARE statements [21].

### Setting

The study was conducted in the largest cities in the UK, Ireland, and the USA: London, Dublin, and New York City, respectively. Participating hospitals were: King’s College Hospital and Guy’s and St Thomas’ Hospital in London; Mater Misericordiae University Hospital and Beaumont Hospital in Dublin; and Mount Sinai Hospital in New York City. All participating hospitals have well-developed specialist palliative care services and are able to refer dying patients to local community hospices (with inpatient beds, day care and community palliative care programs). In addition, Mount Sinai Hospital operates an inpatient palliative care unit within the acute care hospital (Table 1). The London and Dublin hospital palliative care teams are funded and managed by the corresponding National Health Service, which is free at the point of delivery. Palliative care from the New York team is covered by most insurance agencies and Medicare and Medicaid. In all services and settings, additional charitable support is needed for components (Table 1).

**Table 1:** Specialist palliative care service profile in participating cities

	Participating cities		
Specialist palliative care service	London	Dublin	New York
Hospital palliative care team(s) providing a consultative service	Medical Attendings/Consultants, fellows/trainee doctors, clinical nurse specialists/practitioners, psychosocial/social workers, administrative support	Medical Attendings/Consultants, fellows/trainee doctors, clinical nurse specialists/practitioners, psychosocial/social workers, administrative support	Medical Attendings/Consultants, fellows/trainee doctors, clinical nurse specialists/practitioners
Inpatient palliative care unit within service	No	No	Yes, 10 beds
Extended team	Chaplaincy, pharmacy	Pharmacy, physiotherapy, chaplaincy	Social workers, chaplaincy, fellows/trainee doctors, triage nurse
Outpatient clinics	Yes	Yes	Yes
Community palliative care team	Yes (part of catchment area)	No	Yes
On call for specialist advice and emergencies	Yes	Yes	Yes
On call weekend ward rounds	Yes	No	Yes
Co-operation with local hospices^a^	Yes	Yes	Yes
Funding	Paid for by a tariff specifically for activity as part of National Health Service within the hospitals, local inpatient services in hospices paid for by mix of charity and National Health Service. Additional charitable support for components.	Paid for as part of historic block grant allocation as part of National Health Service within the hospitals. Additional charitable support for components.	Program is funded through physician and advance practice nurse billing under Medicare Part B, the Mount Sinai Hospital through Medicare Part A revenues, and contributions from private sector philanthropy.
Annual referrals to services (all ages) at time of study, % >65 years, % with cancer.	1450 Annual referrals (KCH) 70% aged over 65 years 35% Cancer, 65% Non cancer	1370 Annual referrals (MMUH + Beaumont) 74% aged over 65 years 65% Cancer, 35% Non cancer	2175 Annual referrals (consultation service) 57% aged over 65 years 34% Cancer, 66% Non cancer

a Able to refer to external local hospices (i.e., inpatient care and day care, outpatient, community palliative care)

### Procedures

#### Identification and recruitment

We screened consecutive adults accessing specialist palliative care for >24 h. Inclusion criteria were English-speaking and aged ≥65 years. In the first instance, clinicians (usually doctors but also clinical nurse specialists and others caring for patients in palliative care teams) explained the study. If individuals were agreeable to being approached, a researcher fully explained the study to them, provided an information sheet and gained written informed consent. Adults unable to give informed consent or deemed too ill to complete any part of the interview were excluded. This assessment occurred in two stages, first the clinicians (doctors or nurses) in the patients immediate care team were able to indicate if they felt a patient lacked capacity or was too ill or distressed to be approached. In this instance the research team did not approach them. The situation was reviewed after a few days in case any of the issues (such as distress) lessened and patients could then be approached. In a small number of instances clinicians gained approval for the research team to approach patients, but when the researcher visited they deemed that the patient lacked capacity or was too ill to be approached. In these instances, if they felt that the situation might be reversed, they would arrange to re-contact the patient. The process required close working between the research teams and clinicians and considerable flexibility by the researchers. The project employed dedicated research staff to interview patients in their place of choice. Most clinicians raised the study in their regular (usually daily) multidisciplinary team meetings and considered who may be eligible, and agreed which team member would ask the patient. The research team was on hand to quickly approach (usually the same or following day) patients who might be willing to take part. This is essential for very ill populations. Patients had at least 24 h to decide whether to take part in the study. Mental capacity was assessed and if possible improved to allow participation using country-specific guidance. For example in the UK we used, the Mental Capacity Act (MCA) and MCA guidance [22], and equivalent procedures in line with requirements in the US and Ireland, and the MORECARE ethics recommendations [23]. Ethical and data protection approvals do not give consent to collect individual reasons for not being offered the study by clinicians, however local audits indicate that the main reasons were: 1) patients being too unwell as judged by the clinicians or family, usually due to physical symptoms e.g. in severe pain. In these instances if symptoms were resolved individuals were re-approached; 2) cognitive impairment or capacity issue, often because patients were semi or unconscious, or lacked mental capacity due to their illness; and 3) too distressed, as indicated by high levels of anxiety or depression. For symptom distress, if issues improved individuals were re-approached.

#### Questionnaire

The questionnaire was administered in a face-to-face interview with a trained researcher or research nurse. It asked about demographic and clinical information, symptoms and palliative problems in the last 3 days (Palliative care Outcome Scale (POS) [24]), functional status (Barthel Index [25]), cognitive function (Short Orientation Memory and Concentration Test (SOMCT) [26]) and services received. It enquired about priorities and preferences for care, using the format of a major European survey [27, 28].

Questions about most and least preferred place of death were: “If you were in a situation of serious illness with limited time to live, what do you think you would prefer if circumstances allowed you to choose? / What do you think you would least prefer if circumstances allowed you to choose?” Response options were: In your own home, in the home of a relative or friend, in a palliative care unit or hospice (the common term for an inpatient palliative care unit in the UK and Ireland), in a nursing home, in a residential home, somewhere else, don’t know, or refusal/prefer not to say.

Questions were asked about treatment goal priorities and decision making, in a scenario of serious illness. This included asking: “In situations of serious illness with limited time to live difficult decisions may need to be made and some things may need to be prioritised over others. In this situation, would it be more important to:” (three options were given to choose from) extend your life; improve the quality of life for the time you had left; or both are equally important? It also asked:

“Who would you like to make decisions about your care? Please choose as many as apply, you can choose more than one.” YES or NO was given to each of options: yourself, your spouse or partner, other relatives, friends, the doctor, others, don’t know, or refusal/prefer not to say. The questionnaire used in London site is provided (Additional file 1).

Clinical records were reviewed for clinical data; information on adults who died was extracted. Records were reviewed up until end of January 2016. Follow-up ranged from 17 to 39 months from study enrolment.

### Statistical analysis

We calculated summary statistics using proportions and means (standard deviation) and conducted ANOVA tests for differences by city. We examined the distribution of preferences (most and least preferred place of death) by actual place of death and time to death after first referral to specialist palliative care. Count of positive answer to the delegation of decisions on care was compared with the preferences. Our primary dependent variable in regression analyses was whether or not home was the most preferred place of death. Other dependent variables were: least preferred place of death, preference for inpatient hospice/palliative care unit and the actual place of death (to see if these were similar). As an auxiliary examination, we checked the propensity of dying in individuals with different preferences.

We selected potential explanatory variables based on the model developed from results of an international systematic review including over 1.5 million adults, [15] and the availability of the variables: sociodemographic (age, gender, living with others, availability of primary carer, financial status); clinical (diagnosis (cancer or not)), palliative care outcomes (POS total score), functional status (Barthel Index score [25]), cognitive function (SOMCT score [26]); and treatment priorities for quantity and/or quality of life [27]. We used correlations among potential explanatory variables, results from univariate regression analysis and Akaike information criterion (AIC), [29] to choose the final set of explanatory variables for the multivariate regression models [30].

To see if death within 30 days of referral to palliative care was associated with outcome variables, regression analysis was conducted. We also conducted regression analysis with each of the delegation of care decision options, as a dichotomous variable in the model.

Due to the sampling structure (i.e. whether participants from one site e.g. London may have different characteristics to others), we conducted multilevel analysis and used the multivariate logistic regression model with site level fixed effects and a robust variance estimate adjusted for within-site correlation. Our main analysis used complete cases. Missing variables were explored using summary statistics and regression analysis. We conducted two sets of sensitivity analyses. The first set excluded the small number who selected the home of a friend as least preferred while choosing own home as most preferred. The second set used the full information maximum likelihood estimation [31] to impute missing values. STATA version 13.1 was used for all analyses. We estimated that a sample of 130 participants would enable us to enter 10–12 variables in regression analysis. It also enables us to detect a 17% difference between countries in terms of preferences or actual place of death (taking a conservative comparison of 60% v 43% at *p* < 0.05, power 80%), and estimate the proportion with different preferences with a conservative margin of error of <9%.

## Results

### Recruitment and characteristics

We recruited 163 adults: 70 in London, 70 in Dublin and 23 in New York. Thirty-five adults completed only early demographic data and were too ill to complete the latter part of the questionnaire, including the preferences questions. Thus, 138 (64 in London, 59 in Dublin and 15 in New York) were used for the analysis.

Average age was 74 years. New York had more women than London and Dublin. Eighty-eight percent had cancer: genitourinary cancer and digestive cancer was the most common in London, genitourinary cancer in Dublin, digestive cancer in New York. Forty-two percent in London and 59% in Dublin and 53% in New York lived with someone, 76% had a primary caregiver, 35% were living comfortably, 48% coping, and 12% had difficulties on present income (Table 2). Missing data were small and mostly due to illness and/or fatigue. About 8.7% and 5.0% of the sample had missing values on Barthel and SOMCT scales respectively.

**Table 2:** Characteristics of recruited participants (unit: %, mean (sd))

		London	Dublin	New York	All
		(*N* = 64)	(*N* = 59)	(*N* = 15)	(*N* = 138)
Female		47%	44%	80%	49%
Age (years)					
	65–69	31%	34%	33%	33%
	70–74	25%	27%	33%	27%
	75–79	20%	19%	20%	20%
	80–89	16%	17%	13%	21%
	90–96	8%	3%	0%	5%
Diagnosis					
	Lung and respiratory cancer	8%	14%	13%	11%
	Breast cancer	11%	8%	13%	10%
	Genitourinary cancer	25%	27%	7%	24%
	Haematological cancer	8%	3%	20%	7%
	Digestive cancer	25%	24%	27%	25%
	Ill-defined cancer	3%	8%	0%	5%
	Other cancer	8%	2%	13%	6%
	Non-cancer respiratory	3%	2%	0%	2%
	Non-cancer circulatory	5%	7%	0%	5%
	Non-cancer CNS	2%	3%	0%	2%
	Renal failure	0%	0%	7%	1%
	Other non-cancer	3%	2%	0%	2%
	Total Cancer (=1 if cancer)	88%	86%	93%	88%
Marital status					
	Single	13%	14%	20%	14%
	Widowed	31%	36%	13%	31%
	Married/civil partnership	33%	42%	53%	39%
	Divorced/separated	23%	8%	13%	16%
Living with (=1 if with someone else)		42%	59%	53%	51%
Primary carer (=1 if available)		78%	73%	80%	76%
Household income					
	Living comfortably on present	36%	32%	40%	35%
	Coping on present	45%	54%	33%	48%
	Difficult on present	16%	7%	13%	12%
	Very difficult on present	3%	7%	7%	5%
	Prefer not to say	0%	0%	7%	1%
Religion (=1 if religious)		67%	76%	80%	72%
Palliative concerns POS^a^ total score**		13.3 (6.3)	8.1 (5.5)	10.1 (4.7)	10.7 (6.3)
Barthel Index^b^ total score**		69.5 (24.5)	75.8 (26.1)	94.3 (9.6)	75.2 (25.1)
SOMCT^c^ total score		11.3 (5.3)	10.8 (4.4)	7.8 (2.4)	10.8 (4.8)
Participant died as of end January 2016**					
	Yes	86%	88%	20%	80%
	No	9%	2%	53%	11%
	Don’t know	5%	10%	27%	9%
Died within 30 days after referral to palliative care		9%	10%	7%	9%

**p < 0.01 according to the ANOVA tests for differences among the three sites

^a^POS = Palliative care Outcome Scale score, a higher score is worse

^b^Barthel Index score, 0–20 suggests total dependence, 21–60 severe dependence, 61–90 moderate dependence, 91–99 slight dependence and 100 indicates that a patient is independent of assistance from others [25]

^c^SOMCT = Short Orientation Memory and Concentration Test score, a higher score is worse

Participants in New York appeared more functionally independent (mean Barthel score: 94.3) than participants in Dublin (75.8) and London (69.5) (*p* < 0.01; Barthel score 61–90 suggests moderate dependence and 91–99 slight dependence) [25]. Correspondingly, participants in London (SOMCT score: 11.3) and Dublin (10.8) had lower level of cognitive function, compared with participants in New York (SOMCT score: 7.8, *p* < 0.07). Participants in London had more severe symptoms and problems as measured by POS score (13.3) than in New York (10.1) and Dublin (8.1, p < 0.01) (Table 2). Other characteristics did not differ by city. As of end January 2016, 110 participants were confirmed to have died, 15 (11%) were alive; information missing for 13 (9%). Median survival was 146 days.

### Preferences

Most respondents (133/138, 96%) declared preferences regarding treatment priorities, 123/138 (89%) declaring a most preferred place of death and 114/138 (83%) declaring a least preferred place of death.

When asked about treatment priorities in serious illness with limited time to live, only 3% (three people in Dublin, one in London, none in New York) answered that extending their life would be more important than its quality. Most chose one of the other two options, either ‘improving the quality of life for the time left’ (54%) or ‘both extending and quality were equally important’ (39%, but highest in New York: 47%, Table 3).

**Table 3:** Preference for place of death, treatment priorities and decision making

		London	Dublin	New York	All
		(N = 64)	(N = 59)	(N = 15)	(N = 138)
Most preferred place of death^1^					
	Home	42 66%	28 47%	7 47%	77 56%
	Home of a relative or friend	0 0%	0 0%	0 0%	0 0%
	Palliative care unit or inpatient hospice	12 19%	14 24%	5 33%	31 22%
	Hospital	2 3%	4 7%	0 0%	6 4%
	Nursing home or residential home	1 2%	2 3%	0 0%	3 2%
	Elsewhere	4 6%	1 2%	1 7%	6 4%
	Don’t know/prefer not to say	3 5%	10 17%	2 13%	15 11%
Least preferred place of death^2^					
	Home	8 13%	10 17%	4 27%	22 16%
	Home of a relative or friend	9 14%	3 5%	0 0%	12 9%
	Palliative care unit or inpatient hospice	5 8%	1 2%	0 0%	6 4%
	Hospital	18 28%	15 25%	2 13%	35 25%
	Nursing home or residential home	17 27%	6 10%	4 27%	27 20%
	Somewhere else	1 2%	8 14%	2 13%	11 8%
	Don’t know/prefer not to say	6 8%	16 27%	3 20%	25 18%
Treatment goal priority: quantity or quality of life^a3^					
	To extend life	1 2%	3 5%	0 0%	4 3%
	To improve the quality of life for time left	38 59%	30 51%	6 40%	74 54%
	Both are equally important	23 36%	24 41%	7 47%	54 39%
	Don’t know/prefer not to say	2 3%	2 3%	2 13%	5 4%
Person who makes decisions about care^b4^					
	Herself/himself	62 97%	48 81%	10 67%	120 87%
	Spouse or partner	22 34%	17 29%	2 13%	41 30%
	Other relatives	37 58%	31 53%	7 47%	75 54%
	Friends	6 9%	0 0%	1 7%	7 5%
	The doctor	18 28%	11 19%	0 0%	29 21%

*Notes*: ^a^ The exact question used: “*In situations of serious illness with limited time to live difficult decisions may need to be made and some things may need to be prioritized over others. In this situation, would it be more important to extend your life or to improve the quality of life for the time you had left or are both equally important?*”

Statistical test for the difference among countries was conducted using log-likelihood ratio test, adjusting for age, gender and cancer/non-cancer. ^b^ The exact question: “*Who would you like to make decisions about your care? Please choose as many as apply, you can choose more than one.” All but one respondent chose at least one option*

^1^Test for difference: *Χ* ^2^=7.77 (*df* = 14), *p* < 0.9009

^2^ *Χ* ^2^=18.58 (*df* = 18), *p* < 0.4183

^3^ *Χ* ^2^=6.25 (*df* = 8), *p* < 0.6187

^4^ *Χ* ^2^=12.62 (*df* = 2), *p* < 0.0018; *Χ* ^2^=2.04 (*df* = 2), *p* < 0.3609; *Χ* ^2^=1.68 (*df* = 2), *p* < 0.4323; *Χ* ^2^=8.00 (*df* = 2), *p* < 0.0183; *Χ* ^2^=8.57 (*df* = 2), *p* < 0.0138

### Most and least preferred place of death and whether preferences were met

Home was the most common preferred place of death: 56% overall (Table 3). Palliative care unit was the second most preferred (22%). There were no significant differences between cities, but this was highest in New York (33%). The least preferred place for death was in hospital outside of a palliative care unit (25%), followed by nursing or residential home (19%) and own home (16%). Nobody selected the home of a family or friend as their most preferred place of death; six participants (4%) chose their own home as the most preferred place of death and simultaneously another’s home as the least preferred place of death (Additional file 2: Appendix Table A1).

Of the 110 adults who died we were able to confirm place of death for 103. Of these 40 died in a palliative care unit, 35 in hospital, 22 at home and six in a nursing/residential home.

**Fig. 1:**
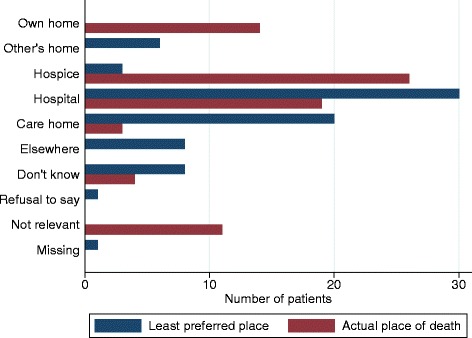
Least preferred and actual place of death for those people who declared they most preferred to die at home (*N* = 77)

Of 62 participants with a known place of death and who had stated their preference was to die at home, 14 (23%) achieved this. Most (26, 42%) died in a palliative care unit. Many died in hospital (19, 31%), often their least preferred place of death (Table 4, Fig. 1). Of the 23 adults who had preferred a palliative care unit, five (23%) achieved this (Table 4). Most of the deaths in palliative care units were people who would have preferred home. Conversely, although 35 older adults died in hospital, this was the preferred place of death for only one (3%). Thirteen adults died at the place which they least preferred, the highest number was in hospital, where eight out of 26 (31%) who had least preferred hospital died there.

**Table 4:** Were preferences met? Actual place of death compared with original preference

	Actual place of death										
Most preferred place of death	Own home	Home of a relative or friend	Palliative care unit or inpatient hospice^a^	Hospital	Care home	Else where	Sub total Known place of death	Don’t know where died^b^	Alive	Total N (%)	
Own home	14	0	26	19	3	0	62	7	4	77	(56%)
Home of a relative or friend	0	0	0	0	0	0	0	0	0	0	(0%)
Palliative care unit or inpatient hospice^a^	7	0	5	10	1	0	23	1	6	31	(23%)
Hospital	0	0	4	1	0	0	5	0	1	6	(4%)
Care home	1	0	1	1	0	0	3	0	0	3	(2%)
Elsewhere	0	0	1	0	1	0	2	1	3	6	(4%)
Don’t know	0	0	1	4	1	0	6	3	1	12	(9%)
Refusal/prefer not to say	0	0	2	0	0	0	2	1	0	3	(2%)
Total	22(21%)	0(0%)	40(39%)	35(34%)	6(6%)	0(0%)	103(100%)	13	15	138	(100%)
Least preferred place of death	Own home	Home of a relative or friend	Palliative care unit or inpatient hospice^a^	Hospital	Care home	Else where	Sub total Known place of death	Don’t know where died^b^	Alive	Total N (%)	
Own home	4	0	5	5	2	0	16	1	5	22	(16%)
Home of a relative or friend	4	0	5	2	1	0	12	0	0	12	(9%)
Palliative care unit or inpatient hospice^a^	0	0	1	1	1	0	3	1	2	6	(4%)
Hospital	5	0	11	8	2	0	26	2	2	33	(24%)
Care home	6	0	7	8	0	0	21	2	4	25	(18%)
Elsewhere	2	0	3	3	0	0	8	1	2	11	(8%)
Don’t know	1	0	5	7	0	0	13	5	0	18	(13%)
Refusal/prefer not to say	0	0	3	0	0	0	3	1	0	4	(3%)
Missing	0	0	0	1	0	0	1	0	0	1	(1%)
Total	22(21%)	0(0%)	40(39%)	35(34%)	6(6%)	0(0%)	103(100%)	13	15	138	(100%)

The exact questions used in the questionnaire were: *“If you were in a situation of serious illness with limited time to live… A. Where do you think you would prefer to die if circumstances allowed you to choose? B. So which of these do you think you would least prefer if circumstances allowed you to choose?”*

^a^The hospital in New York has a palliative care unit, which is a specially adapted ward. ^b^Not able to ascertain place of death

A palliative care unit was rarely least preferred (by only six (4%) respondents, Table 3). Of the 40 people who died in a palliative care unit, it was least preferred for only one (Table 4).

### Factors associated with home as the most preferred place of death

Participants with cancer (OR 3.72, 95% CI 1.40–9.90) or living with somebody else (OR 2.19, 95% CI 1.33–3.62) were more likely to prefer their home as a place of death to other places. Adults with higher Barthel scores of functional independence were marginally less likely to prefer home as a place of death (OR 0.99, 95% CI 0.98–1.00; note CI reaches 1 due to rounding). Age was not associated with any most preferred place of death. When we asked about the least preferred place of death, non-cancer was associated with home as a least preferred place of death (OR 4.29, 95% CI 1.23–15.00) (Table 5). Sensitivity analysis, excluding those who chose another’s home as the least preferred choice, found the same results, except that effects of cancer was statistically significant (cancer patients were less likely to least prefer home).

**Table 5:** Multivariate logistic regression: factors associated with a preference for home or inpatient hospice/palliative care unit

		Home as the most preferred			Home as the least preferred			Palliative care unit or inpatient hospice as the most preferred		
		Odds ratio	CI _Lower_	CI _Upper_	Odds ratio	CI _Lower_	CI _Upper_	Odds ratio	CI _Lower_	CI _Upper_
Female		0.53	0.11	2.51	1.81	0.93	3.51	1.68	0.21	13.87
Age (base: 65–69)^a^										
	70–79	0.95	0.13	6.82	1.48	0.56	3.91	1.30	0.11	14.81
	80–96	0.77	0.49	1.23	1.71	0.66	4.47	1.79	0.40	8.05
Cancer		**3.72** ^**^	**1.40**	**9.90**						
Non-cancer					**4.29** ^*^	**1.23**	**15.00**	**2.39** ^*^	**1.14**	**5.03**
Living with		**2.19** ^**^	**1.33**	**3.62**	1.02	0.32	3.23			
Barthel total score		**0.99** ^**^	**0.98**	**1.00**	1.03	0.98	1.07	1.00	1.00	1.01
28-SOMCT total score								**1.09** ^*^	**0.97**	**1.23**
Constant		**2.42** ^**^	**1.86**	**3.13**	**0.01** ^*^	**<0.00**	**0.52**	**0.02** ^**^	**<0.00**	**0.25**
N			113			102			107	
Log likelihood			−67.16			−45.71			−54.92	

^**^ *p* < 0.01 and ^*^ *p* < 0.05. Standard errors are adjusted for 3 clusters in Site. Site level fixed effects model was estimated

^a^Joint test for age categories was: *Χ* ^2^ = 3.18 (*df* = 2), *p* < 0.2.43; *Χ* ^2^ = 1.21 (*df* = 2), *p* < 0.5471; *Χ* ^2^ = 5.89 (*df* = 2), *p* < 0.0527

### Factors associated with palliative care unit (inpatient hospice) as the most preferred place of death

Participants with non-cancer diagnosis (OR 2.39, 95% CI 1.14–5.03) or higher SOMCT scores (OR 1.09, 95% CI 0.97–1.23) were more likely to choose palliative care unit/inpatient hospice as the most preferred place of death (Table 5).

### Propensity of mortality among samples

Having cancer was associated with dying sooner, but confidence intervals were wide (OR 9.3, 95% CI 1.5–57.8). There were no consistent patterns of other factors associated with dying sooner in sensitivity analysis. (Additional file 2: Appendix Table S4). Those preferring death at home were no different in time to death than those who preferred elsewhere (Additional file 2: Appendix Table S2 and Additional file 2: Figure S1).

### Factors associated with actual place of death

No sample from New York was used in this analysis due to small numbers. Age, diagnosis and living status were not significant in the analysis. Higher functional independence (OR 1.05, 95% CI 1.04–1.06) was associated with greater likelihood of dying at home. Participants who valued only quality of life (OR 3.11, 95% CI 2.89–3.36) were more likely to die at home than those who valued both quality and extension of life (Additional file 2: Appendix Table S5).

### Relationships with delegation of decisions on care

87% positively answered they would like to make decisions on care, 30% for spouse or partner, 54% for other relatives, 5% for friends and 21% for the doctors themselves (Table 3). In regression analysis, wanting a spouse/partner to make decisions was associated with preference for home (OR 1.92 95% CI 1.42–2.59); opting for friends was associated with preference for palliative care unit/hospice (OR 4.05 95% CI 1.24–13.18); and choosing a spouse/partner or friends with actual home death (OR 2.84 95% CI 1.83–4.40; OR 4.00 95% CI 1.96–8.14) (Additional file 2: Appendix Table S3).

## Discussion

In this first multi-centre study of the preferences of older adults with advanced disease across in three countries, home was consistently the most preferred place and hospital the least preferred place, with no differences between countries adjusting for age, gender and cancer diagnosis. In the USA, patients had the highest preference for hospital palliative care units – an option not commonly available to those in the UK or Ireland, where the closest option is usually an inpatient hospice. Having a cancer diagnosis and living with someone else were independently associated with preferring to die at home. A preference for involving partners or spouses in healthcare decisions was also associated with preferring home. In contrast, diagnosis and living status were not associated with place of death, and instead functional status and preferring treatments aimed to improve quality of life were associated with dying at home.

Our data shed light on the more nuanced patterns of choice around the end of life. Home appears to be a polarizing choice; it is definitely the choice of a majority, but also interestingly the least favoured option of around 16%. Not having cancer was independently associated with choosing home as least preferred. Reasons for this may be related to perceived burden to others, [32] wishing to feel safe, [11] failings in care continuity or coordination [33] and the quality of support at home, especially out-of-hours support [11, 19]. Further work is needed to understand whether not wanting to die at home is a positive choice, or a reaction against failures in care [19]. Choice is complicated by cognitive biases, such as forecasting errors and default options, that pervade human decision making [34]. For example, in these choices, being unaware of, or having unrealistic forecasts about the nature of home or hospital or palliative care or the nature of the problems they or their family may face are likely to influence the preferences expressed. Individuals may also report the choice that they think is the most socially acceptable to their family or friends or which they think is the most common (default option). They may also express the preference that they think will leave them with the most remaining options should that option fail. Data on the quality of care is vital here, as home is only able to be a choice if services meet individual needs and are of good quality. As Shakespeare said in *The Taming of the Shrew* “There's small choice in rotten apples.” Feeling unsafe at home is emerging as a leading reason for people to seek care in hospital [35].

Our study is the first to show that the home of a relative or family member should not be considered as an equal alternative to patients dying in their own home. We found a family member’s home was never the most preferred, and was least preferred for almost 1 in 10. Thus, dying in the house of family members or friends should not be promoted as an alternative to dying at home. Preferences for home are thought to be related to a wish for familiar surroundings, flexible regime and control [7]. The home of a family member may well not meet these requisites, as well as leading to greater fear of being a burden.

Our study found that general hospital wards were definitely disliked by many: these were very rarely a preferred choice and the least preferred for most. Similarly, nursing homes were rarely favoured. Inpatient palliative care units and hospices emerged as an important alternative to home; these were the second most common preference after home, and seem to be relatively acceptable as only 6 people (4%) considered these the least preferred option. We were interested to see the high proportion from the USA favouring this option. In the USA, as for the USA site in this study, palliative care units are often designated wards for the care of patients receiving palliative care within hospitals. Thus, a palliative care unit or inpatient hospice within a hospital appears to be a viable alternative, at least as far as choice is considered. People with non-cancer conditions were more likely to choose this option. This diverges from provision in many countries: people with non-cancer conditions have low access to inpatient hospice or palliative care units [36–39]. We found that most people who wanted to die at home actually died in a palliative care unit or inpatient hospice. Indeed the group wanting to die at home formed the majority dying in a hospice or palliative care unit. Those wanting home were more likely to die in a palliative care unit or hospice than those people who had actually wanted to die in that setting. This may be because many hospices prioritise transfers from home, rather than from hospitals, and suggests that the availability of inpatient hospice and palliative care beds needs expansion. The cost effectiveness of home and inpatient palliative care services is now being established, further supporting these initiatives [40, 41]. With such services the actual place of death can be more often at home and in palliative care units, [42, 43] which our study suggests would more closely meet patient choice.

In our study, while just over 50% of older patients preferred to die at home, patients’ deaths mainly occurred elsewhere, with different factors associated with preferences and reality. Interventions that may help address this disparity include advance care planning, as older adults involved in some form of advance care planning may be less likely to die in a hospital (adjusted relative risk (aRR) = 0.87, 95% CI 0.80–0.94) [44]. National studies in both the UK and USA have also found that hospital admission is driven by distressing symptoms [45] with inadequate pain management for older people in primary care [46] and nursing homes [47]. Trials of cost-effective ways to alleviate symptoms and provide high quality and safe care for older adults with advanced disease in the community 24/7 are therefore urgently needed.

Our finding of greater functional independence being associated with a home death contradicts findings from a major systematic review, although the review included only those who died from cancer [15]. Adults with higher levels of functional independence may feel that they are less of a burden on their families. In addition, those with more need for physical care may place a greater demand on community resources, driving hospital admission [45]. However, we found a divergence from factors associated with preferences, as those with higher functional independence were marginally less likely to prefer home.

Taken together these findings suggest that use of ‘meeting’ a preference as a quality indicator is limited. Across all three countries the variation between most and least preferred suggests that any quality indicator, rather than focussing on meeting individual preferences alone, should consider whether individual ‘least favoured’ preferences were avoided, and should understand influencing factors. It is vital also to assess the capacity to meet preferences in that setting, including the quality of care, wherever care is offered even when measurement is challenging [48]. The quality of care can include controlling pain and symptoms and addressing concerns, information and practical and financial issues, such as measured in the Palliative care Outcome Scale [49, 50]. It may also include financial hardship, such as out of pocket expenses [51]. Recent research has found an increase in emergency department use during the last year of life, despite falling hospital deaths, for people with both cancer [52] and dementia, [53] suggesting that quality indicators need to consider a longer period of time than only the point of death. The use of population-based quality indicators, such as avoiding hospitalisation, and care in and dying at home or in an inpatient palliative care unit, are supported by our results which found consistent patterns in these preferences. Their use is valid at a population level, rather than at an individual level as some aspects such as home is clearly not favoured by an important proportion of the population, although it is consistently the majority wish in all setting and by all groups. There remains a marked gap between the proportion that wish to be cared for at home or in an inpatient palliative care unit and those who eventually die there, indicating that more resources are needed in these settings.

A major strength of our study is that we successfully included those with advanced diseases. Most research about preferences is on the general population, whose views may change following illness [7]. However, we may have missed other individuals not receiving palliative care, some of whom may not have wanted such care, and consequently may have different preferences. While the nature of questions meant that those with cognitive impairment were excluded from the study, this also makes the results not generalizable to all older people. Few older people with cognitive impairment, for example with dementia, die in specialist palliative care units, and the majority die in long-term care facilities or in acute hospitals [54]. Eighty-eight percent of participants had a cancer diagnosis, most likely because the sample was from those receiving specialist palliative care, although the focus of specialist palliative care is changing and encompasses more conditions. However, our results may have a skewed view of preferences. It is well documented that those dying from dementia and other chronic diseases have similar end of life symptoms as those dying from cancer, although with a different trajectory. Although our non cancer group was small, it is important to note that non cancer was associated with a preference for in-patient hospice and palliative care, despite this usually being little available. It may be that in cases other than cancer, it is only those individuals with very strong expressed preferences for hospice and palliative care who can gain access. Further work is needed among other broader samples of older people with advanced illness, especially in non specialist palliative care settings. If possible this should employ proxy or other methods to understand preferences when people have cognitive impairment.

We specifically asked individuals if they wished not to state a preference, and/or if they did not know, and have reported these data in our overall percentages, as recommended [55]. Eleven percent of respondents opted not to state a most preferred place of death, and 18% least preferred. A King’s Fund population based survey found that 75% of people said choice was either ‘very important’ or ‘important’ to them, [56] which would be consistent with our results. In the King’s Fund survey, older respondents were more likely to value choice if they were with no qualifications and from a mixed or non-white background [56]. More work is needed to understand whether particular groups elect ‘no preference’, whether a preference would have emerged over time, whether it did not matter to respondents, or whether respondents did not want to discuss the issue with the interviewer. Finally, not all patients had died at the point of follow-up, and so are missing from the place of death analysis.

## Conclusions

Home and palliative care unit were the most commonly preferred place of death in all countries. In this sample, having cancer and living with others are associated with home preference. A non-cancer diagnosis was associated with preference for a palliative care unit. Different factors were associated with place of death than with preferences. We propose that actual place of death and its fit to the needs and preferences of patients and their families should be measured when assessing the quality of end of life care. Available services need to be improved to more closely meet actual needs, for example by providing the 24/7 level of community palliative care support needed to enable home deaths, and further developing inpatient palliative care units (in hospitals or freestanding hospices) while ensuring access for those with non-malignant conditions. The home of a relative or family member or a care home should not be considered as an equal alternative to a person’s own home.

## Additional files

Additional file 1: Copy of the questionnaire. Questionnaire used in the study for IARE project. Note this is British version of the questionnaire. There were minor amendments to some introductory text in USA and Dublin (e.g. opening explanation), to comply with local requirements, but the questions remained the same. (PDF 477 kb)

Additional file 2: Determinants choices appendices tables and figures. Appendices tables and figures in numerical order. Appendices tables and figures referred to in the main manuscript text. (DOC 185 kb)

## Abbreviations

ANOVA: The ANalysis Of Variance
CLAHRC: Collaboration for Leadership in Applied Health Research and Care
IARE: International Access, Rights and Empowerment Programme (the name of this project used in the BuildCARE Programme, which appears on the questionnaires)
MORECARE: Methods Of Researching palliative and End of life CARE: development of best practice guidance
POS: Palliative (Patient) care Outcome Scale
SOMCT: Short Orientation Memory and Concentration Test
STATA: a general-purpose STATistical software package created by StataCorp
STROBE: STrengthening the Reporting of OBservational studies in Epidemiology
