# Supplementary material for: Social and clinical determinants of preferences and their achievement at the end of life: prospective cohort study of older adults receiving palliative care in three countries
Source: BMC Geriatr. 2017 Nov 23;17:271. doi: 10.1186/s12877-017-0648-4 (PMC5701500; doi:10.1186/s12877-017-0648-4)
Supplement: Supplementary file 1 — Copy of the questionnaire. Questionnaire used in the study for IARE project. Note this is British version of the questionnaire. There were minor amendments to some introductory text in USA and Dublin (e.g. opening explanation), to comply with local requirements, but the questions remained the same. (PDF 477 kb) [file 12877_2017_648_MOESM1_ESM.pdf]

Document Title: Face-to-face survey including an example of the choice experiment schedule

Research Ethics Committee Reference Number: 12/LO/0044

'Version 3 29/10/2012'

International Access, Rights and Empowerment Study (IARE)

Project BuildCARE

Face-to-face patient survey

Introduction and screening (patient)

"Hello, may I speak to [PATIENT'S NAME]?

My name is [YOUR NAME]. I am working on a study with patients receiving palliative care services. The goal of the study is to better understand the needs of patients and their caregivers during this difficult time. We are doing this by speaking to individuals such as yourself who can provide important information about your health needs, the care received and the costs associated with the care provided.

This information will help improve care for other patients and families in the future.

I realize that this is a difficult time for you, but I wonder if I might ask you some questions. Is this a good time for us to talk? It should not take us more than 60 minutes."

[DISCUSSION TO INCLUDE AS NECESSARY: INFORMATION SHARING, GENERAL AND SPECIFIC QUESTIONS AND ANSWERS REGARDING STUDY, EMPHASISE RIGHT TO REFUSE, NO INFLUENCE ON TREATMENT, CARE OPTIONS, EXPLAIN WITHDRAWAL OPTIONS]

[CONSENT PROCESS BEGINS]

[ARRANGE TIME FOR INTERVIEW AND AGREE UPON SITE]

DEMOGRAPHIC INFORMATION:

ID No		Hospital	

Please circle the correct answer or write the correct answer in the relevant box:

Age (years)		Gender	Male	Female

Marital Status	Single	Widowed	Married/in a civil partnership/ relationship akin to marriage	Divorced/Separated

Lives Alone	Yes	No

(If answered no) Who do you live with?	With Children	With Spouse/Partner	With Friend/s	With other unspecified

[Apart from the health workers, do you have someone who helps care for you? Try to help them establish who their primary carer might be: for example is it their: wife/husband or partner; son or daughter; brother or sister; parent; other relative (specify); friend or neighbour; staff in nursing or residential home; warden in sheltered housing; other official (specify). If they say yes they do have others caring for them then help them answer the following question. If they don't identify anyone then please write "no primary carer".]

Primary carer? Yes or No

Relationship of Carer to patient	

Educational level	Did not go to school	Secondary School (GCSE)	Secondary School (A-Levels)	Post Secondary School Vocational Qualifications	University

Main Occupation	

Postal District of residence	

[Introductory question: Do you work in paid or unpaid employment?]

Current Annual Income in the last year (include income from pension)	Nil or loss	£1 to £3,999	£4,000 to £7,999	£8,000 to £11,999	£12,000 to £16,999	£17,000 to £23,999	£24,000 to £36,999	£37,000 or more

Which of the following descriptions comes closest to how you feel about your household's income nowadays?
Answer options: (one answer option only)

☐ Living comfortably on present income
☐ Coping on present income
☐ Difficult on present income
☐ Very difficult on present income

[NOT TO BE READ, JUST FOR THE INTERVIEWER TO SEE AND TICK IF APPLICABLE]?

☐ Don't know
☐ Refusal/prefer not to say

What is your ethnic group? PROBE, CODE ONLY ONE – *Choose one section from A to E, then tick one box to best describe the ethnic group or background.*

A. White

☐ English /Welsh / Scottish / Northern Irish / British
☐ Irish
☐ Gypsy or (Irish) Traveller
☐ Any other White background (write in) ________

B. Mixed / multiple ethnic groups

☐ White and Black Caribbean
☐ White and Black African
☐ White and Asian
☐ Any other Mixed / multiple ethnic background (write in) ___________

C. Asian / Asian British

☐ Indian
☐ Pakistani
☐ Bangladeshi
☐ Chinese
☐ Any other Mixed / multiple ethnic background (write in) ___________

D. Black / African / Caribbean / Black British

☐ African
☐ Caribbean
☐ Any other Black / African / Caribbean background (write in) __________

E. Other ethnic group

☐ Arab

[NOT TO BE READ, JUST FOR THE INTERVIEWER TO SEE AND TICK IF APPLICABLE]

☐ Don't know
☐ Refusal/prefer not to say

Do you consider yourself as belonging to any particular religion or denomination? PROBE. CODE ONLY ONE.

☐ Yes - **CONTINUE**
☐ No – **SKIP TO PALLIATIVE CARE OUTCOME SCALE**

[NOT TO BE READ, JUST FOR THE INTERVIEWER TO SEE AND TICK IF APPLICABLE]
☐ Don't know
☐ Refusal/prefer not to say

If yes, which one? **PROBE. CODE ONLY ONE.**

☐ Roman Catholic
☐ Church of England / Anglican
☐ Church or Ireland
☐ Baptist
☐ Methodist
☐ Presbyterian / Church of Scotland
☐ United Reform Church/Congregational
☐ Free Presbyterian
☐ Brethren
☐ Other Protestant (WRITE IN) ____________________
☐ Greek or Russian Orthodox
☐ Other Eastern Orthodox (WRITE IN) ______________
☐ Other Christian (WRITE IN) _____________________
☐ Hindu
☐ Sikh
☐ Buddhist
☐ Other Eastern Religions (WRITE IN) ______________
☐ Jewish
☐ Islam / Muslim
☐ Other non-Christian (WRITE IN) __________________
☐ Other (specify) _____________________

Palliative care Outcome Scale (version two)

Thank you. Now, I would like to ask some specific questions about your general well-being. Please answer the following questions by telling us which option you think most accurately describes how you have been feeling in the past 3 days.

**1**	**Over the past 3 days, have you been affected by pain?**<br><br>0 Not at all, no effect<br>1 Slightly – but not bothered to be rid of it<br>2 Moderately – pain limits some activity<br>3 Severely – activities or concentration markedly affected<br>4 Overwhelmingly – unable to think of anything else	
**2**	**Over the past 3 days, have other symptoms e.g. feeling sick, having a cough or constipation been affecting how you feel?**<br><br>0 No, not at all<br>1 Slightly<br>2 Moderately<br>3 Severely<br>4 Overwhelmingly	
**3**	**Over the past 3 days, have you been feeling anxious or worried about your illness or treatment?**<br><br>0 No, not at all<br>1 Occasionally<br>2 Sometimes – affects my concentration now and then<br>3 Most of the time – often affects my concentration<br>4 Can’t think of anything else - completely pre-occupied by worry and anxiety	
**4**	**Over the past 3 days, have any of your family or friends been anxious or worried about you?**<br><br>0 No, not at all<br>1 Occasionally<br>2 Sometimes – it seems to affect their concentration<br>3 Most of the time<br>4 Yes, always preoccupied with worry about me	
**5**	**Over the past 3 days, how much information have you and your family or friends been given?**<br><br>0 Full information – always feel free to ask what I want<br>1 Information given but hard to understand<br>2 Information given on request but would have liked more<br>3 Very little given and some questions were avoided<br>4 None at all	

**6**	**Over the past 3 days, have you been able to share how you are feeling with your family or friends?**<br><br>0 Yes, as much as I wanted to<br>1 Most of the time<br>2 Sometimes<br>3 Occasionally<br>4 Not at all with anyone	
**7**	**Over the past 3 days, have you been feeling depressed?**<br><br>0 No, not at all<br>1 Occasionally<br>2 Sometimes<br>3 Most of the time<br>4 Yes, all the time<br>*If you have placed a tick in boxes 3 or 4 for this question, please speak with your nurse or doctor at your next appointment.*	
**8**	**Over the past 3 days, have you felt good about yourself as a person?**<br><br>0 Yes, all the time<br>1 Most of the time<br>2 Sometimes<br>3 Occasionally<br>4 No, not at all	
**9**	**Over the past 3 days, how much time do you feel has been wasted on appointments relating to your healthcare, e.g. waiting around for transport or repeating tests?**<br><br>0 None at all<br>2 Up to half a day wasted<br>4 More than half a day wasted	
**10**	**Over the past 3 days, have any practical matters resulting from your illness, either financial or personal, been addressed?**<br><br>0 Practical problems have been addressed and my affairs are as up to date as I would wish<br>2 Practical problems are in the process of being addressed<br>4 Practical problems exist which were not addressed<br>0 I have had no practical problems	
**11**	**If any, what have been your main problems in the last 3 days?**<br><br>1 …………………………………………………………………………………………………….<br>2 …………………………………………………………………………………………………….	
**12**	**Any other comments**	
	**Total Score (Items 1-10 only)**	

Condensed Memorial Symptom Assessment Scale – symptoms

Thank you. Shall we stop for a break or are you OK to proceed?

This set of questions will ask you about some symptoms which you may or may not have experienced.

If you have the symptom, please indicate "Yes". If you indicated "Yes", please tell us how much the symptom has bothered or distressed you in the past 7 days.

**Symptom**	Present	Not at all	A little bit	Some what	Quite a bit	Very much
Lack of energy	Y / N	0	1	2	3	4
Lack of appetite	Y / N	0	1	2	3	4
Pain	Y / N	0	1	2	3	4
Dry mouth	Y / N	0	1	2	3	4
Weight loss	Y / N	0	1	2	3	4
Feeling drowsy	Y / N	0	1	2	3	4
Shortness of breath	Y / N	0	1	2	3	4
Constipation	Y / N	0	1	2	3	4
Difficulty sleeping	Y / N	0	1	2	3	4
Difficulty concentrating	Y / N	0	1	2	3	4
Nausea	Y / N	0	1	2	3	4

Below are other commonly listed symptoms. Please indicate if you have had the symptom in the **past 7 days**. If so, how often did they occur?

**Symptom**	Present	Rarely	Occasionally	Frequently	Almost constantly
Worrying	Y / N	1	2	3	4
Feeling sad	Y / N	1	2	3	4
Feeling nervous	Y / N	1	2	3	4

Barthel Index – activities of daily living

Again thank you, we have now nearly finished the survey. Are you OK to continue or shall we stop for a break?

The next series of questions refer to your ability to perform day–to-day activities.

Please tell us which activity score which best applies to you. A score of 0 indicates that you are unable to perform the activity, anything more than zero indicates some ability. A score of 10 indicates that you are fully able to do the activity without help.

Activity Score

**FEEDING**<br>0 = unable<br>5 = needs help cutting, spreading butter, etc., or requires modified diet<br>10 = independent	
**BATHING**<br>0 = dependent<br>5 = independent (or in shower)	
**GROOMING / PERSONAL CARE**<br>0 = needs help with personal care<br>5 = independent face/hair/teeth/shaving (implements provided)	
**DRESSING**<br>0 = dependent<br>5 = needs help but can do about half unaided<br>10 = independent (including buttons, zips, laces, etc.)	
**BOWELS**<br>0 = incontinent (or needs to be given enemas)<br>5 = occasional accident<br>10 = continent	
**BLADDER**<br>0 = incontinent, or catheterized and unable to manage alone<br>5 = occasional accident<br>10 = continent	
**TOILET USE**<br>0 = dependent<br>5 = needs some help, but can do something alone<br>10 = independent (on and off, dressing, wiping)	

**TRANSFERS (BED TO CHAIR AND BACK)**	
0 = unable, no sitting balance<br>5 = major help (one or two people, physical), can sit<br>10 = minor help (verbal or physical)<br>15 = independent	
**MOBILITY (ON LEVEL SURFACES)**	
0 = immobile or < 50 yards<br>5 = wheelchair independent, including corners > 50 yards<br>10 = walks with help of one person (verbal or physical) > 50 yards<br>15 = independent (but may use any aid; for example, stick) > 50 yards	
**STAIRS**	
0 = unable<br>5 = needs help (verbal, physical, carrying aid)<br>10 = independent	
**TOTAL (0–100):**	

FAMCARE

Thank you. In this section of the survey, I would like to ask you some questions about the care that you have received during your stay here at the hospital (or your last hospital stay) **[ONLY SAY "LAST HOSPITAL STAY" IF PATIENT BEING INTERVIEWED AT HOME]**. Please tell me how satisfied you are with the care received: very satisfied (VS), satisfied (S), undecided (U), dissatisfied (D) or very dissatisfied (VD).

[Interviewer to circle the letters below that best match the experience].

How satisfied are you with:

1	Pain relief	VS	S	U	D	VD
2	Information provided about your prognosis	VS	S	U	D	VD
3	Answers from health professionals	VS	S	U	D	VD
4	Information given about side effects	VS	S	U	D	VD
5	Referrals to specialists	VS	S	U	D	VD
6	Availability of a hospital bed	VS	S	U	D	VD
7	Family conferences (or conferences that involve friends or others that care for you) held to discuss your illness	VS	S	U	D	VD
8	Speed with which symptoms are treated	VS	S	U	D	VD
9	Doctor's attention to your description of symptoms	VS	S	U	D	VD
10	The way tests and treatments are performed	VS	S	U	D	VD
11	Availability of doctors to your family or others involved in your care e.g. friends	VS	S	U	D	VD
12	Availability of nurses to your family or others involved in your care e.g. friends	VS	S	U	D	VD
13	Coordination of care	VS	S	U	D	VD
14	Time required to make a diagnosis	VS	S	U	D	VD
15	The way your family or others (e.g. friends) are included in treatment and care decisions	VS	S	U	D	VD
16	Information given about how to manage your pain	VS	S	U	D	VD
17	Information given about your tests	VS	S	U	D	VD
18	How thoroughly the doctor assesses your symptoms	VS	S	U	D	VD
19	The way tests and treatments are followed up by the doctor	VS	S	U	D	VD
20	Availability of the doctor to you	VS	S	U	D	VD

Comments (interviewer to record any comments that people wish to make about above or other aspects of care here):

....

....

....

....

Short Orientation Memory and Concentration Test

Before we begin the next section of the questionnaire about your preferences for care, I'd like to ask a few general questions:

Instruction

0-28 points; a higher score means greater disability. Score 1 error for each incorrect response.

**Items**	**Questions**	**Score**	**Weight**		**Score**
1.	What year is it now?<br>*(score 1 if incorrect; score 0 if correct)*		x 4	=	
2.	What month is it now?<br>*(score 1 if incorrect; score 0 if correct)*		x 3	=	
Memory phrase	Address : Repeat this address after me: John Brown, 42 West Street, Gateshead<br>**Try to remember this. I'll ask you to recall it at the end of the test.**				
3.	Can you tell me what time it is? (within one hour)<br>*(score 1 if incorrect; score 0 if correct)*		x 3	=	
4.	Count backwards from 20 to 1<br>20 19 18 17 16 15 14 13 12 11 10 9 8 7 6 5 4 3 2 1<br>*(Score 1 if 1 error; Score 2 if 2 or more errors. A maximum of 2 errors is allowed; Score 0 if correct)*		x 2	=	
5.	Say the months of the year in reverse order<br>Dec, Nov, Oct, Sept, Aug, July, June, May, April, March, Feb, Jan<br>*(Score 1 if 1 error; Score 2 if 2 or more errors. A maximum of 2 errors is allowed; Score 0 if correct)*		x 2	=	
6.	Can you repeat the address again for me?<br>John / Brown / 42 / West Street / Gateshead<br>*(Score 1 for every error; A maximum of 5 errors is allowed; Score 0 if correct)*		x 2	=	

Total score: /28

[Choice experiment component]

In this part of the survey we wish to find out your preferences for specialist palliative care regardless of where you may receive their input.

We will present you with two options and ask you to choose the option you most prefer.

We may present you with less ideal options at times. We appreciate it might be hard to make a choice when given these less than ideal options but we encourage you to choose because all of your choices will help us to understand what you preferences are.

We will continue to present the choices until we have presented you with every available option and identified your choices.

All of the options we will give to you are about the palliative care team.

This part of the survey will be recorded and I'll make some notes as we go along. All of your information will be treated confidentially and anonymously.

Any questions?

Are you ready to begin?

Alright, I'll turn the recording on now. **[TURN RECORDING DEVICE ON NOW]**

Here's the first set of options, and remember this is about your preferences for palliative care.

[THE OPTION IS PRESENTED HERE BY THE INTERVIEWER]

Please choose the option that best represents your choice – option A or option B.

[END OF CHOICE EXPERIMENT]

We have now come to the end of the choice experiment.

I'm turning the recording device off now. **[TURN RECORDING DEVICE OFF]**

Thank you. We are now entering into the final section of the survey. There are four more questions to go. They are a little bit more sensitive in nature. This section aims to find out what your preference would be in a hypothetical scenario or situation. If you would rather not answer you are free to do so.

Are you OK to continue with the next section?

If you were in a situation of serious illness with limited time to live...

A. Where do you think you would prefer to die <u>if circumstances allowed you to choose</u>?
[WAIT FOR THE RESPONDENT TO ANSWER AND THEN CODE THE ANSWER AS PER THE OPTIONS BELOW. IF THE ANSWER IS UNABLE TO BE CODED THROUGH THE OPTIONS PROVIDED IN THE LIST PROVIDED THEN PLEASE CLARIFY FURTHER TO IDENTIFY THEIR PREFERENCE SO THAT IT TO BE CODED ACCURATELY]
CODE ONLY ONE.

B. **So which of these do you think you would least prefer <u>if circumstances allowed you to choose</u>?**
READ OUT LIST OF ANSWER OPTIONS WITH PAUSES BETWEEN EACH. REPEAT IF NECESSARY. CODE ONLY ONE.

	A	B
**Answer options**	**Most preferred**	**Least preferred**
In your own home		
In the home of a relative or friend		
In a hospice or palliative care unit - places with specialised care and beds for dying patients		
In hospital - but not in a palliative care unit		
In a nursing home		
In a residential home		
Somewhere else (please say where): ________________		
**NOT TO BE READ, JUST FOR THE INTERVIEWER TO SEE AND TICK IF APPLICABLE**		
Don't know		
Refusal/prefer not to say		

In situations of serious illness with limited time to live difficult decisions may need to be made and some things may need to be prioritised over others. **In this situation, would it be more important to extend your life or to improve the quality of life for the time you had left or are both equally important?**

Answer options (one option only):

☐ To extend life
☐ To improve the quality of life for the time you had left
☐ Both are **<u>equally</u>** important
[NOT TO BE READ, JUST FOR THE INTERVIEWER TO SEE AND TICK IF APPLICABLE.]
☐ You don't know
☐ Refusal/prefer not to say

Again in a scenario of serious illness, please consider now that you were able to make decisions.

Who would you like to make decisions about your care? Please choose as many as apply, you can choose more than one. READ OUT EACH OPTION ONE BY ONE AND GET A YES / NO OPTION TO EACH BEFORE MOVING ON TO THE NEXT OPTION. CODE ALL THAT APPLY.

☐ Yourself
☐ Your spouse or partner
☐ Other relatives
☐ Friends
☐ The doctor
☐ Others (**PLEASE SPECIFY/PROBE**): ________________________
[NOT TO BE READ, JUST FOR THE INTERVIEWER TO SEE AND TICK IF APPLICABLE.]
☐ Don't know
☐ Refusal/prefer not to say

What if you had lost your ability to make decisions, who would you like to make decisions about your care? Please choose as many as apply, you can choose more than one.

READ OUT. CODE ALL THAT APPLY.
☐ Yourself, by specifying your wishes before losing ability - for example, in a living will
☐ Your spouse or partner
☐ Other relatives
☐ Friends
☐ The doctor
☐ Others (**PLEASE SPECIFY/PROBE**): ________________________
[NOT TO BE READ, JUST FOR THE INTERVIEWER TO SEE AND TICK IF APPLICABLE.]
☐ Don't know
☐ Refusal/prefer not to say

Thank you for answering these questions and for your contribution to this research. Your answers have been really helpful and will help improve the care that others receive including carers.

We have now come to the end of the interview.

Is there anything else you would like to share before we finish?

If anything we have discussed today has been emotionally difficult for you, I could ask one of the clinicians to meet with you. Would you like me to speak to one of them on your behalf? Alternatively I could find a number for a helpline so that you could talk to someone. Would you like this number?

Thank you again for your help with this survey. We hope to publish the results from our work.

Would you like to receive any information regarding the study?

Would you like to receive a summary or copy of the results?

Your answers were helpful and will be used to help improve care to others including caregivers.

Thank you. [END OF FACE-TO-FACE SURVEY]
