# Supplementary material for: Social and clinical determinants of preferences and their achievement at the end of life: prospective cohort study of older adults receiving palliative care in three countries
Source: BMC Geriatr. 2017 Nov 23;17:271. doi: 10.1186/s12877-017-0648-4 (PMC5701500; doi:10.1186/s12877-017-0648-4)
Supplement: Supplementary file 2 — Determinants choices appendices tables and figures. Appendices tables and figures in numerical order. Appendices tables and figures referred to in the main manuscript text. (DOC 185 kb) [file 12877_2017_648_MOESM2_ESM.doc]

**Additional file**

***Social and clinical determinants of preferences and their achievement at the end of life: prospective cohort study of older adults receiving palliative care in three countries***.

| **Table S1. Most Preferred Place of Death versus Least Preferred Place of Death** | | | | | | | | | | | | |
| --- | --- | --- | --- | --- | --- | --- | --- | --- | --- | --- | --- | --- |
|  |  | Least preferred place of death | | | | | | | | |  | |
| Most preferred place of death | | Own home | Home of  a relative or friend | Palliative care unit or inpatient hospice^a^ | Hospital | Care home | Else  where | Don’t know | Prefer not to say | Missing | Total | |
| Own home | | 0 | 6 | 3 | 30 | 20 | 8 | 8 | 1 | 1 | 77 | 56% |
| Home of a relative or friend | | 0 | 0 | 0 | 0 | 0 | 0 | 0 | 0 | 0 | 0 | 0% |
| Palliative care unit or inpatient hospice^a^ | | 11 | 6 | 0 | 2 | 7 | 1 | 4 | 0 | 0 | 31 | 23% |
| Hospital | | 5 | 0 | 0 | 0 | 0 | 0 | 1 | 0 | 0 | 6 | 4% |
| Care home | | 2 | 0 | 0 | 0 | 0 | 0 | 1 | 0 | 0 | 3 | 2% |
| Elsewhere | | 3 | 0 | 1 | 0 | 0 | 1 | 1 | 0 | 0 | 6 | 4% |
| Don't know | | 1 | 0 | 2 | 3 | 0 | 1 | 5 | 0 | 0 | 12 | 9% |
| Prefer not to say | | 0 | 0 | 0 | 0 | 0 | 0 | 0 | 3 | 0 | 3 | 2% |
| Total | | 22 16% | 12 9% | 6 4% | 35 25% | 27 20% | 11 8% | 20 15% | 4 3% | 1 1% | 138 100% | 100% |

The exact questions used in the questionnaire were: *“If you were in a situation of serious illness with limited time to live… A. Where do you think you would prefer to die if circumstances allowed you to choose? B. So which of these do you think you would least prefer if circumstances allowed you to choose?”*

^a^The hospital in New York has a palliative care unit, which is a specially adapted ward.

| **Table S2. Preference for Place of Death and Time to Death after Referral to Specialist Palliative Care (N=138)** | | | | | | | | |
| --- | --- | --- | --- | --- | --- | --- | --- | --- |
|  | |  | Time to death after referral | | | Alive | Death information missing | Total |
|  | |  | ≤ 30 days | > 30 days | Missing^b^ |
| Most preferred place of death | | |  |  |  |  |  |  |
|  | Home (own) | | 8  62% | 53  59% | 5  71% | 4  27% | 7  58% | 77  56% |
|  | Palliative care unit or inpatient hospice | | 2  15% | 21  23% | 1  14% | 6  40% | 1  8% | 31  23% |
|  | Other | | 3  23% | 16  18% | 1  14% | 5  33% | 5  39% | 30  22% |
|  | Sub total | | 13  100% | 90  100% | 7  100% | 15  100% | 13  100% | 138 |
| Least preferred place of death | | |  |  |  |  |  |  |
|  | Home (own & other’s) | | 2  15% | 26^a^  29% | 0  0% | 5  33% | 1  8% | 34  25% |
|  | Palliative care unit or inpatient hospice | | 0  0% | 3  3% | 0  0% | 2  13% | 1  8% | 6  5% |
|  | Other | | 11  85% | 61  68% | 7  100% | 8  53% | 11  85% | 98  71% |
|  | Sub total | | 13  100% | 90  100% | 7  100% | 15  100% | 13  100% | 138 |

^a^14 patients chose own home and 12 patients chose home of a relative or friend.

^b^Due to missing on referral date and/or date of death.

**Table S3**. Delegation of care decisions and preference

|  |  | Who would you like to make decisions about your care | | | | |
| --- | --- | --- | --- | --- | --- | --- |
| Most preferred place of death |  | Self  (n=120) | Spouse / partner  (n=41) | Other relatives  (n=75) | Friends  (n=7) | The doctor  (n=29) |
| Own home | 77 | 71 | 27 | 40 | 4 | 20 |
|  | 56% | 59% | 66% | 53% | 57% | 69% |
| Home of a relative or friend | 0 | 0 | 0 | 0 | 0 | 0 |
|  | 0% | 0.0% | 0.0% | 0.0% | 0.0% | 0.0% |
| Palliative care unit or inpatient hospice^a^ | 31 | 27 | 6 | 21 | 3 | 4 |
|  | 23% | 23% | 15% | 28% | 43% | 14% |
| Hospital | 6 | 4 | 0 | 3 | 0 | 1 |
|  | 4% | 3% | 0% | 4% | 0% | 3% |
| Care home | 3 | 1 | 1 | 1 | 0 | 0 |
|  | 2% | 1% | 2% | 1% | 0% | 0% |
| Elsewhere | 6 | 6 | 1 | 1 | 0 | 2 |
|  | 4% | 5% | 2% | 1% | 0% | 7% |
| Don't know | 12 | 8 | 4 | 7 | 0 | 2 |
|  | 9% | 7% | 10% | 9% | 0% | 7% |
| Refusal/prefer not to say | 3 | 3 | 2 | 2 | 0 | 0 |
|  | 2% | 3% | 5% | 3% | 0% | 0% |
| Total |  | 120 | 41 | 75 | 7 | 29 |
| Least preferred place of death |  |  |  |  |  |  |
| Own home | 28 | 16 | 4 | 11 | 0 | 4 |
|  | 20% | 13% | 10% | 15% | 0% | 14% |
| Home of a relative or friend | 12 | 12 | 2 | 7 | 2 | 2 |
|  | 9% | 10% | 5% | 9% | 29% | 7% |
| Palliative care unit or inpatient hospice^a^ | 6 | 6 | 2 | 2 | 1 | 0 |
|  | 4% | 5% | 5% | 3% | 14% | 0% |
| Hospital | 33 | 31 | 14 | 18 | 1 | 10 |
|  | 24% | 26% | 34% | 24% | 14% | 34% |
| Care home | 25 | 25 | 6 | 14 | 2 | 10 |
|  | 18% | 21% | 15% | 19% | 29% | 34% |
| Elsewhere | 11 | 10 | 4 | 7 | 0 | 1 |
|  | 8% | 8% | 10% | 9% | 0% | 3% |
| Don't know | 18 | 15 | 6 | 12 | 1 | 2 |
|  | 13% | 13% | 15% | 16% | 14% | 7% |
| Refusal/prefer not to say | 4 | 4 | 3 | 3 | 0 | 0 |
|  | 3% | 3% | 7% | 4% | 0% | 0% |
| Missing | 1 | 1 | 0 | 1 | 0 | 0 |
|  | 1% | 1% | 0% | 1% | 0% | 0% |
| Total | 138 | 120 | 41 | 75 | 7 | 29 |

The exact questions used in the questionnaire were: *“Who would you like to make decisions about your care? Please choose as many as apply, you can choose more than one.”*

^a^The hospital in New York has a palliative care unit, which is a specially adapted ward.

| **Table S4. Multivariate Logistic Regression: Factors Associated with Death Among Samples** | | | | |
| --- | --- | --- | --- | --- |
|  | | Death as of January 2016 | | |
|  | | Odds ratio | CI _Lower_ | CI _Upper_ |
| Female | | **0.12^**^** | **0.06** | **0.22** |
| Age (base: 65-69)^§^ | |  |  | |
|  | 70-79 | 0.09 | 0.00 | 1.66 |
|  | 80-96 | 0.20 | 0.00 | 8.60 |
| Household income (base: Living comfortably on present income^¶^ | |  |  | |
|  | Coping on present income | **0.74** | **0.13** | **4.11** |
|  | Difficult on present income | **0.36** | **0.16** | **0.79** |
| Cancer | | **9.27^*^** | **1.49** | **57.81** |
| Living with | | 0.38 | 0.07 | 2.18 |
| SOMCT total score | | 1.07 | 1.01 | 1.21 |
| Constant | | 39.83 | 0.93 | 1710.54 |
| N | |  | 113 |  |
| Log likelihood | |  | -20.46 |  |

^**^p<0.01 and ^*^p<0.05. Standard errors are adjusted for 3 clusters in Site. Site level fixed effects model was estimated.

§ Joint test for overall effect: *Χ*^2^=2.63 (*df*=2), *p*<0.2679.

¶ Joint test for overall effect: *Χ*^2^=7.90 (*df*=2), *p*<0.0193

| **Table S5. Multivariate Logistic Regression: Factors Associated with Actual home Death** | | | | | | |  |
| --- | --- | --- | --- | --- | --- | --- | --- |
|  | | Home death (Model 1) | | | Home death (Model II) | | |
|  | | Odds ratio | CI _Lower_ | CI _Upper_ | Odds ratio | CI _Lower_ | CI _Upper_ |
| Preference for home death | |  |  |  | 0.50 | 0.09 | 2.92 |
| Female | | 1.22 | 0.11 | 13.02 | 1.27 | 0.14 | 11.30 |
| Age (base: 65-69)^§^ | |  |  | |  |  |  |
|  | 70-79 | 1.28 | 0.62 | 2.65 | 1.50 | 0.67 | 3.39 |
|  | 80-96 | 0.58 | 0.22 | 1.55 | 0.49 | 0.19 | 1.26 |
| Household income (base: Living comfortably on present income^¶^ | |  |  | |  |  |  |
|  | Coping on present income | 0.49 | 0.13 | 1.85 | 0.47 | 0.10 | 2.25 |
|  | Difficult on present income | 2.30 | 1.39 | 3.83 | 1.72 | 0.89 | 3..34 |
| Barthel total score | | **1.05^**^** | **1.04** | **1.06** | **1.05^**^** | **1.04** | **1.06** |
| Treatment goal priority (base: Extension and quality of life) | |  |  |  |  |  |  |
|  | Improvement in quality of life | **3.11^**^** | **2.89** | **3.36** | **3.75^**^** | **3.54** | **3.97** |
| Constant | | **0.00^**^** | **0.00** | **0.01** | **0.01^**^** | **0.00^**^** | **0.03** |
| N | |  | 79 |  |  | 72 |  |
| Log likelihood | |  | -32.03 |  |  | -29.84 |  |

^**^p<0.01. Standard errors are adjusted for 2 clusters in Site. Site level fixed effects model was estimated.

No observation in New York was used in this analysis.

In sensitivity analysis using full information maximum likelihood to impute the missing information, effects were smaller (e.g. OR 1.13, 95% CI 1.10–1.15 for *Improvement in quality of life*).

§ Joint test for overall effect: *Χ*^2^=1.18 (*df*=1), *p*<0.2778 for Model I and *Χ*^2^=2.20 (*df*=1), *p*<0.1377 for Model II.

¶ Joint test for overall effect: *Χ*^2^=1.10 (*df*=1), *p*<0.2944 for Model I and *Χ*^2^=0.91 (*df*=1), *p*<0.3414 for Model II.

**Figure S1. Survival by the Most Preferred Place of Death (Home or Other).**

Note: Observations with missing information on the preference for place of death, referral date and/or date of death were excluded. χ2=2.39, p>0.12.
